# Cytoskeletal Control of Planar Polarity in Root Hair Development

**DOI:** 10.3389/fpls.2020.580935

**Published:** 2020-09-04

**Authors:** Hirotomo Takatsuka, Masaki Ito

**Affiliations:** School of Biological Science and Technology, College of Science and Engineering, Kanazawa University, Kanazawa, Japan

**Keywords:** root hair, planar polarity, cytoskeleton, actin, microtubule, Rho-of-plant, *Arabidopsis thaliana*

## Introduction

Polarity is at the core of plant development, as well as that of other multicellular organisms since polarization acts as a driving force to generate a variety of specialized cells. Therefore, unraveling how cells establish polarity is the most fundamental issue toward understanding the principles of plant morphogenesis.

Cell polarity coordinated within the plane of a single tissue layer is particularly termed “planar polarity (also termed planar cell polarity),” which offers an experimentally accessible model system. Therefore, planar polarity is studied in various multicellular organisms. In the animal kingdom, polarized arrangement of ommatidia and bristles in *Drosophila melanogaster* and the orientation of hair follicles in mammalian skin are well-known examples of planar polarity.

## Root Hair Positioning as a Model System for Planar Polarity

**Figure 1:**
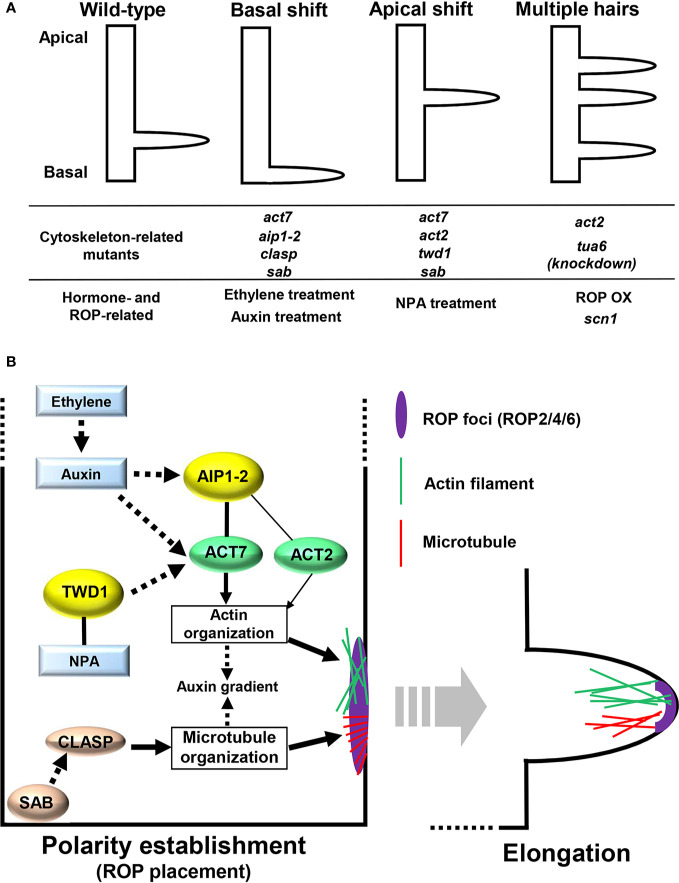
**(A)** Root hair positioning of wild-type, mutants with altered cytoskeleton or Rho-of-plants (ROP) level, and plants treated with reagents related to auxin and ethylene. **(B)** Cytoskeletal control of root hair positioning. Black solid lines represent direct protein interactions. Black solid and black broken arrows represent direct or indirect action on the target, respectively.

Root hairs possess crucial roles in nutrient and water uptake from the soil, as well as in providing anchorage for plants (Gilroy and Jones, 2000). In *Arabidopsis thaliana*, root hair emergence is restricted to close to the basal ends (rootward ends) of specialized cells aligned within a single file, namely the hair-forming cell lineage (trichoblasts), thus manifesting typical planar polarity (**Figure 1A**) (Masucci and Schiefelbein, 1994; Fischer et al., 2007). The site of root hair emergence intriguingly appears to be evolutionarily conserved across plant species (Salazar-Henao et al., 2016; Dolan, 2017) and is easily observable under laboratory conditions without any special equipment; root hair positioning provides an excellent model to elucidate the mechanisms behind establishing polarity in plants, which indeed has been studied extensively.

## Rho-of-Plants, an Early Marker for Root Hair Positioning

The members of the Rho-of-plant (ROP) small GTPase protein family are implicated in establishing root hair planar polarity. Among the 11 members of the ROP family in *Arabidopsis*, ROP2, ROP4, and ROP6 localize to the hair initiation site (**Figure 1B**) (Molendijk et al., 2001; Jones et al., 2002). Severe defects in root hair growth were observed in the *rop2/4/6* triple mutant (Gendre et al., 2019). More importantly, increased ROP activities from either overexpression of *ROP2* or defects in ROP-GDP dissociation inhibitor lead to root hair formation at multiple sites in one cell (**Figure 1A**) (Jones et al., 2002; Carol et al., 2005), indicating that an adequate ROP protein level needs to be placed at the adequate site to establish planar polarity during root hair development. ROP proteins begin accumulating at the future hair initiation site prior to the actual onset of hair formation (**Figure 1B**) (Molendijk et al., 2001), providing an early marker for the established root hair planar polarity; thus, clarifying the mechanism behind ROP placement at the future initiation site is key toward understanding planar polarity establishment during root hair development.

## Auxin and Ethylene Provide a Cue for Root Hair Positioning

Among phytohormones, auxin and ethylene have been reported to provide a cue for root hair positioning (Masucci and Schiefelbein, 1994; Vicente-Agullo et al., 2004). Exogenous application of either auxin or 1-aminocyclopropane-1-carboxylic acid (ACC), an ethylene precursor, causes a basal shift of root hair positioning. Root hairs in plants with either a reduced level of auxin signaling or ethylene perception emerge from more apical sites compared with those in wild-type plants (**Figure 1A**) (Masucci and Schiefelbein, 1994; Ikeda et al., 2009). Ikeda et al. (2009) reported that the auxin applied locally at the very tip of roots was actively transported to hair-forming cells, eventually generating a basal shift of root hair positioning. Contrastingly, auxin transport inhibition from the root tip to hair-forming cells results in an apical shift (**Figure 1A**). These findings suggest that auxin redistribution involving both local synthesis and polar transport is required for properly establishing root hair planar polarity. Ethylene potentially acts upstream of auxin to control planar polarity by regulating local auxin synthesis (**Figure 1B**) (Ikeda et al., 2009). Recent studies have shed light on the role of the cytoskeleton in mediating hormonal control of root hair positioning,

## Cytoskeletal Control of the ROP Placement in Root Hair Initiation

Eukaryotic cells possess three types of cytoskeleton, namely, actin microfilaments (MFs), microtubules (MTs), and intermediate filaments; the presence of intermediate filaments in plants is albeit still unresolved (Kost and Chua, 2002; Ivakov and Persson, 2013). In this review, we describe recent advances in actin- and MT-mediated establishment of root hair planar polarity.

### Actin

The involvement of the actin cytoskeleton in root hair positioning has long been recognized, according to the finding that a mutation in the *ACTIN2* (*ACT2*) gene encoding globular actin (G-actin) causes an apical shift of root hair positioning in *Arabidopsis* (**Figure 1A**) (Ringli et al., 2002). Moreover, the knockout mutant for *ACT7*, another member of the *ACT* gene family in *Arabidopsis*, exhibits more severe defects in root hair positioning than *act2*; both apical and basal shifts of root hair positioning are frequently observed in *act7* (**Figure 1A**) (Kiefer et al., 2015). Filamentous actin (F-actin) formed through polymerization of G-actin encoded by *ACT* genes is highly accumulated at the future initiation site before polar root hair emergence takes place; hence, actin filaments containing G-actin produced from *ACT7* and/or *ACT2* are possibly required for establishing planar polarity in root hair development (**Figure 1B**) (Kiefer et al., 2015). Recent studies identified key regulators that modulate ROP placement and eventual root hair positioning by influencing actin dynamics.

Kiefer et al. (2015) found that ACT7 and ACT2 modulate planar polarity along with their direct interactor, ACTIN-INTERACTING PROTEIN1-2 (AIP1-2) (**Figure 1B**). The *aip1-2* knockout mutant displays a basal shift of the ROP placement and resultant root hair positioning as in *act* mutants (**Figure 1A**). Moreover, analyses of the genetic interaction between *ACT7* and *AIP1-2* revealed that AIP1-2 acts upstream of ACT7 (**Figure 1B**) (Kiefer et al., 2015). AIP1-2 affects actin-filament organization by physically interacting with ACT7, thereby determining ROP placement (**Figure 1B**). Ethylene and auxin signaling possibly converges on the AIP1-2-ACT7 module to control root hair planar polarity (**Figure 1B**), since expression of *ACT7* and *AIP1-2* is upregulated by auxin and ethylene in an auxin-dependent manner, respectively (Kandasamy et al., 2001; Kiefer et al., 2015). However, whether the AIP1-2-ACT7 module precisely determines the ROP placement site is still unclear.

TWISTED DWARF1 (TWD1) is another actin-related key regulator for root hair planar polarity (**Figure 1B**) (Zhu et al., 2016). TWD1 is an FK506-binding protein originally identified as a direct interactor of N-1-naphthylphthalamic acid (NPA), an auxin-transport inhibitor (**Figure 1B**) (Geisler et al., 2003; Bailly et al., 2008), and *twd1* mutant showed an apical shift of root hair positioning, resembling NPA treatment of wild-type roots (**Figure 1A**) (Zhu et al., 2016). TWD1 interacts with ACT7, albeit indirectly, to remodel actin organization (**Figure 1B**) (Zhu et al., 2016). The TWD1-mediated control of actin organization has been shown to influence auxin redistribution in roots by modulating the subcellular localization of both ABCB- and PIN-type auxin transporters (Zhu et al., 2016). Therefore, the TWD1-ACT7 module possibly mediates root hair planar polarity by controlling auxin transport (**Figure 1B**). However, in the absence of clear evidence, it still remains possible that abnormally organized actin filaments in *twd1* are responsible for root hair misplacement, independent of the perturbed auxin transport.

Myosin, a motor protein involved in vesicle trafficking by interacting with actin filaments, is required for tip growth of root hair, based on the finding that the lack of a member of the myosin family results in a shorter root hair phenotype (Park and Nebenführ, 2013). However, whether actin-myosin interaction is required for ROP placement and root hair positioning remains elusive.

### Microtubule

Microtubules (MTs), which consist of α-tubulin and β-tubulin, play an essential role in determining plant cell shape, including that of root hair (Hashimoto, 2015). Impaired MT organization induced by MT-depolymerizing drugs, such as oryzalin, gives rise to shorter and wavy root hairs (Bibikova et al., 1999). In comparison, the knowledge concerning their role in root hair positioning is limited; however, accumulated evidence indicates their involvement in establishing root hair planar polarity. Firstly, MTs are dynamically reorganized at the future hair initiation site; transversely aligned cortical MTs are rearranged into unique radial patterns (**Figure 1B**) (Pietra et al., 2013). Furthermore, Bao et al. (2001) demonstrated that a reduction in α-tubulin leads to multiple root hair formation from one cell, reminiscent of ROP overexpressors (**Figure 1A**), implying the role of MTs in proper ROP placement. Lastly, the engagement of the MT-associated protein CLIP170-ASSOCIATED PROTEIN (CLASP) and its genetic interactor SABRE (SAB) in establishing root hair planar polarity has been recently reported (**Figure 1B**) (Pietra et al., 2013). CLASP, known as a central regulator of cell-division plane orientation, is required for properly determining ROP placement; *clasp* mutant exhibits a basal shift of ROP foci (**Figure 1A**). SAB, with an unknown molecular function, localizes to the plasma membrane and regulates cortical MT dynamics (Pietra et al., 2013). Unlike *clasp*, root hair positioning shifts either apically or basally in *sab* (**Figure 1A**). Moreover, *sab clasp* double mutant is indistinguishable from *sab*, suggesting that *sab* is epistatic to *clasp* in controlling planar root hair polarity (Pietra et al., 2013). CLASP has been notably reported to regulate the abundance of PIN2 protein, which is a central auxin transporter in root epidermis, by regulating MT dynamics, thereby playing an important role in auxin gradient formation (**Figure 1B**) (Ambrose et al., 2013). It is still unclear whether CLASP-SAB-guided MT reorganization directly sets ROP proteins at the proper site or indirectly influences ROP placement by controlling auxin transport (**Figure 1B**).

Kinesins are motor proteins that assist cells with transport of molecules along microtubules. The armadillo domain-containing putative kinesin MRH2 has been reported to control root hair morphology by altering MT organization (Yang et al., 2007), but kinesin involvement in root hair positioning is still unclear.

## Summary and Future Perspectives

Root hair positioning provides an excellent model for planar polarity, but some important questions and points are still open as follows: 1) how does the cytoskeleton place ROPs at the future initiation site? The cytoskeleton possibly acts as a scaffold of ROP proteins, constraining ROP subcellular localization at the initiation site. To support this view, in some cases, the cytoskeleton affects polarity establishment by functioning as a scaffold-like structure required for polarization of key molecules (Inagaki and Katsuno, 2017; Liu et al., 2018; Raman et al., 2018). For example, in leaf epidermal cells, actin filaments are implicated in PIN protein recycling to the plasma membrane, enabling polarized PIN localization (Xu et al., 2010). Recently, Denninger et al. (2019) found that the accumulation of ROPGEF3, which tethers and activates ROPs at the plasma membrane, at the future hair initiation site precedes that of ROPs; therefore, it is possible that the cytoskeleton places ROPGEF3 at the future hair site and thereby determines the ROP position. 2) The direct link between auxin signaling and the cytoskeleton is still missing. Despite extensive studies on the target genes of auxin response factors (ARFs), a transcription factor family acting downstream of auxin signaling, there is still no evidence showing that ARF directly regulates cytoskeleton-related genes involved in root hair planar polarity (Okushima et al., 2005; Schlereth et al., 2010; Boer et al., 2014). Note that it remains possible that auxin affects cytoskeletal organization through non-transcriptional responses, as is the case of leaf cells wherein auxin alters cytoskeletal dynamics in a manner that does not require ARF-dependent transcriptional responses, thereby forming zigzag-shaped pavement cells (Xu et al., 2010). 3) In many tissues, actin filaments and MTs cooperatively regulate many aspects of plant morphogenesis, including root hair elongation (Takeuchi et al., 2017). However, their cooperative actions have not been elucidated in terms of root hair planar polarity. 4) Small GTPases other than ROPs might participate in establishing root hair polarity. Indeed, a recent study demonstrated that ARF-GAP that localizes to the hair initiation is required for root hair positioning (Yoo et al., 2018). However, whether and how the cytoskeleton is engaged in the placement of other small GTPases is still unknown. The answers to these important questions will help to better understand more general principle of how polarity is established in plant cells.

## Author Contributions

All authors listed have made a substantial and direct contribution to the work, and approved it for publication.

## Funding

This work was supported by JSPS KAKENHI Grant Number 19K05951 and the 2017 Inamori Research Grant Program to HT.

## Conflict of Interest

The authors declare that the research was conducted in the absence of any commercial or financial relationships that could be construed as a potential conflict of interest.
